# Role of dimerization and palmitoylation on the function of HTLV-1 p12 and p8

**DOI:** 10.1186/1742-4690-8-S1-A124

**Published:** 2011-06-06

**Authors:** Dustin Edwards, Risaku Fukumoto1, Nancy Van Prooyen, Heather Gold, Maria F de Castro-Amarante, Luiz Alcantara, Genoveffa Franchini

**Affiliations:** 1 Animal Models and Retroviral Vaccines Section, National Cancer Institute, National Institutes of Health, Bethesda, Maryland, 20892, USA

## 

HTLV-1 p12 is an endoplasmic reticulum resident protein that is cleaved to generate p8, which traffics to the cell surface. p12 promotes T-cell activation by increasing NFAT activity while p8 induces T-cell anergy and enhances virus transmission. We have previously demonstrated that p12 and p8 coimmunoprecipitate. Though the dimerization domain of p12 and p8 is unknown, these proteins contain a single cysteine residue which may form intermolecular disulfide bonds. We have also determined that p8 localizes to membrane lipid rafts. Of importance, palmitoylation increases the hydrophobicity of proteins to target them to lipid rafts. As palmitoylation occurs on cysteine residues, we hypothesize that this post-translational modification regulates p12 and p8 localization, dimerization, and function. In this study, we have demonstrated that wildtype p12 and p8 formed hetero- and homodimers while a mutation at the cysteine residue (C39A) inhibited dimer formation and that monomeric wildtype p12 and p8, but not the C39A mutant, are pamitoylated. Immunofluorescence analysis showed that wildtype p8 localized at the cell surface while C39A p8 did not. This result suggests that p8 homodimerization or palmitoylation regulates p8 localization. We also analyzed ex vivo DNA samples from HTLV-1-infected individuals and found DNA polymorphisms at position 39. These naturally occurring polymorphisms affected dimerization and localization of p8. Currently, we are investigating whether mutation at C39 affects NFAT activation, virus transmission, or proviral load. Determining the mechanism by which p12 and p8 localization, dimerization, and functions are regulated will add to our understanding of HTLV-1 pathogenesis.

